# Seaweed consumption and prevalence of depressive symptoms during pregnancy in Japan: Baseline data from the Kyushu Okinawa Maternal and Child Health Study

**DOI:** 10.1186/1471-2393-14-301

**Published:** 2014-09-03

**Authors:** Yoshihiro Miyake, Keiko Tanaka, Hitomi Okubo, Satoshi Sasaki, Masashi Arakawa

**Affiliations:** Department of Public Health, Ehime University Graduate School of Medicine, Toon, Ehime Japan; Department of Preventive Medicine and Public Health, Faculty of Medicine, Fukuoka University, Fukuoka, Japan; Department of Health Promotion, National Institute of Public Health, Saitama, Japan; Department of Social and Preventive Epidemiology, Graduate School of Medicine, The University of Tokyo, Tokyo, Japan; Health Tourism Research Center, Graduate School of Tourism Sciences, University of the Ryukyus, Okinawa, Japan

**Keywords:** Cross-sectional study, Depressive symptoms, Intake, Japanese women, Pregnancy, Seaweed

## Abstract

**Background:**

Seaweed is a popular traditional food in Japan and is a rich source of bioactive metabolites. The neuroprotective properties of seaweed have attracted attention; to date, however, there has been no epidemiological evidence regarding the relationship between seaweed consumption and depression. The current cross-sectional study investigated the association between seaweed consumption and depressive symptoms during pregnancy in Japan.

**Methods:**

Study subjects were 1745 pregnant women. Depressive symptoms were defined as present when subjects had a Center for Epidemiologic Studies Depression Scale score of 16 or higher. Dietary consumption during the preceding month was assessed using a self-administered diet history questionnaire. Adjustment was made for age; gestation; region of residence; number of children; family structure; history of depression; family history of depression; smoking; secondhand smoke exposure at home and at work; job type; household income; education; body mass index; and intake of fish and yogurt.

**Results:**

The prevalence of depressive symptoms during pregnancy was 19.3%. After adjustment for possible dietary and non-dietary confounding factors, higher seaweed consumption was independently associated with a lower prevalence of depressive symptoms during pregnancy: the adjusted odds ratios (95% confidence intervals) for depressive symptoms during pregnancy in the first, second, third, and fourth quartiles of seaweed consumption were 1 (reference), 0.72 (0.51 − 1.004), 0.71 (0.50 − 1.01), and 0.68 (0.47 − 0.96), respectively (*P* for trend = 0.03).

**Conclusions:**

The present results suggest that seaweed consumption may be inversely associated with the prevalence of depressive symptoms during pregnancy in Japanese women.

## Background

Seaweed has been a very important part of the Japanese diet for many centuries and is an important source of fiber, minerals, vitamins, polysaccharides, and iodine. Seaweed is a rich source of bioactive metabolites, and compounds found in it have various biological activities including anticoagulant, anti-viral, antioxidant, anti-allergic, anti-cancer, anti-inflammatory, anti-obesity, and neuroprotective properties [1]. Nevertheless, epidemiological information concerning the effects of seaweed consumption has been scarce. Inverse associations have been reported between seaweed consumption and stomach cancer [2], colorectal cancer [3], allergic rhinitis [4], mortality from all causes, pancreatic cancer, lung cancer, kidney cancer, and cerebrovascular disease [5], breast cancer [6], and elevated blood pressure [7], while positive associations have been shown between seaweed consumption and prostate cancer [8], upper aerodigestive tract cancer [9], mortality from colon cancer [5], metabolic syndrome [10], and thyroid cancer [11].

Several lines of laboratory studies have provided insight into the neuroprotective effects of seaweed, suggesting that seaweed consumption might affect depressive symptoms in humans [1]. To our knowledge, there has been no epidemiological evidence regarding the relationship between seaweed consumption and depression. The current cross-sectional study aimed to investigate the association between seaweed consumption and depressive symptoms during pregnancy in Japanese women using baseline data from the Kyushu Okinawa Maternal and Child Health Study (KOMCHS).

## Methods

### Study population

The KOMCHS is an ongoing prospective prebirth cohort study that examines risk and preventive factors for maternal and child health problems. Details of the baseline survey of the KOMCHS have been described elsewhere [12]. Eligible women were those who became pregnant while living in one of seven prefectures on Kyushu Island in southern Japan, with a total population of approximately 13.26 million, or in Okinawa Prefecture, an island chain in the southwest of Japan, with a total population of nearly 1.37 million. Between April 2007 and March 2008, we requested that 423 obstetric hospitals in the above-mentioned eight prefectures provide as many pregnant women as possible with a set of leaflets explaining the KOMCHS, an application form to participate in the study, and a self-addressed and stamped return envelope. Pregnant women who were willing to participate in the KOMCHS returned the application form containing a written description of their personal information to the data management center. Upon receiving this personal information, research technicians gave each participant a detailed explanation of the KOMCHS by telephone and sent them a self-administered questionnaire after obtaining their agreement. In total, 1757 pregnant women between the 5th and 39th weeks of pregnancy gave their written informed consent to participate in the KOMCHS and completed the baseline survey. Excluded were 12 pregnant women with missing data on the variables under study; thus, data on 1745 pregnant women were used for analysis. The KOMCHS was conducted according to the guidelines laid down in the Declaration of Helsinki, and all procedures involving human subjects were approved by the ethics committee of the Faculty of Medicine, Fukuoka University. The STROBE (Strengthening The Reporting of Observational studies in Epidemiology) guidelines were followed.

### Measurements

In the baseline survey, each participant filled out a two-part questionnaire and mailed it to the data management center between the 5th and 39th week of pregnancy. Research technicians clarified or completed missing or unclear data by telephone.

The first part of the questionnaire collected information on age; gestation; region of residence; number of children; family structure; personal history of doctor-diagnosed depression; family history of depression; smoking habits; secondhand smoke exposure at home and at work; employment status; household income; and educational level. A family history of depression was considered to be present if one or more parents or siblings of the study subjects had been diagnosed with depression by a physician. Employment status was ascertained for the year when the baseline survey was conducted and the previous year; women were classified as being unemployed if they were unemployed both in the year when the baseline survey was conducted and in the preceding year.

Depressive symptoms were assessed by means of a Japanese version [13] of the Center for Epidemiologic Studies Depression Scale (CES-D) [14], which was incorporated into the first part of the questionnaire. This scale consists of 20 questions addressing six symptoms of depression, including depressed mood, feelings of guilt or worthlessness, helplessness or hopelessness, psychomotor retardation, loss of appetite, and sleep disturbance experienced during the preceding week. Each question is scored on a scale of 0 to 3 according to the frequency of the symptoms, and the total CES-D score ranges from 0 to 60. The criterion validity of the CES-D scale has been well established in adult Western [14] and Japanese [13] populations. Following the validation study, we defined depressive symptoms as present when a subject had a CES-D score ≥ 16.

The second part of the questionnaire was a semi-quantitative, comprehensive diet history questionnaire (DHQ) that assessed dietary habits during the preceding month [15, 16]. Estimates of daily consumption of foods (total of 150 foods), energy, and selected nutrients were calculated using an ad hoc computer algorithm for the DHQ based on the Standard Tables of Food Composition in Japan [17]. Seaweed consumption was calculated using semiquantitative frequency questions regarding two separate food items: Wakame or Hijiki and Nori. For each of the two food items, consumption frequency was estimated in terms of eight categories (ranging from “two or more times per day” to “less than once a month”), and relative portion size was estimated in terms of five categories: very small (50% or less), small (about 70-80%), medium (about 100%), large (about 20-30% larger), and very large (50% larger or more) of a standard portion size. Energy-adjusted consumption calculated according to the residual method was used for the analyses [18]. Body weight and height were self-reported as part of the DHQ. Body mass index was calculated as weight (kg) divided by the square of height (m).

### Statistical analysis

Seaweed consumption was categorized at quartile points on the basis of the distribution of all study subjects. Age; gestation; region of residence; number of children; family structure; history of depression; family history of depression; smoking; secondhand smoke exposure at home and at work; job type; household income; education; body mass index; and consumption of fish and yogurt were selected *a priori* as potential confounding factors. Higher consumption levels of fish [12] and yogurt [19] were significantly associated with a lower prevalence of depressive symptoms during pregnancy in this population. Age, gestation, body mass index, and dietary confounding factors were used as continuous variables.

Logistic regression analysis was used to estimate crude odds ratios (ORs) and 95% confidence intervals (CIs) of depressive symptoms during pregnancy in relation to the quartile of seaweed consumption, with the lowest quartile as the reference. Multiple logistic regression analysis was used to adjust for potential confounding factors. Trend of association was assessed according to a logistic regression model assigning consecutive integers (1 to 4) to the quartiles of seaweed consumption. All computations were performed using the SAS software package version 9.2 (SAS Institute, Inc., Cary, NC, USA).

## Results

The prevalence of depressive symptoms during pregnancy was 19.3% among the 1745 pregnant women. The mean age of the subjects and gestation at baseline were 31.2 years and 18.5 weeks, respectively (Table 1). About 5% of the subjects had a personal history of depression and 10% had a family history of depression. Mean daily total energy consumption and mean daily energy-adjusted seaweed consumption during pregnancy were 7434.2 kJ and 12.3 g, respectively.

**Table 1:** **Distribution of selected characteristics in 1745 pregnant women**

Variable	***n***(%)
Age, years, mean ± SD	31.2 ± 4.3
Gestation, weeks, mean ± SD	18.5 ± 5.4
Region of residence	
Fukuoka Prefecture	971 (55.6)
Other than Fukuoka Prefecture in Kyushu	592 (33.9)
Okinawa Prefecture	182 (10.4)
Number of children	
0	703 (40.3)
1	690 (39.5)
≥ 2	352 (20.2)
Nuclear family structure	1474 (84.5)
History of depression	84 (4.8)
Family history of depression	175 (10.0)
Having ever smoked	563 (32.3)
Ever experiencing secondhand smoke exposure at home	1315 (75.4)
Ever experiencing secondhand smoke exposure at work	1106 (63.4)
Job type^a^	
Unemployed	705 (40.4)
Professional or technical	435 (24.9)
Clerical or related occupation	328 (18.8)
Sales	83 (4.8)
Service	115 (6.6)
Production	51 (2.9)
Others^b^	28 (1.6)
Household income, yen/year	
< 4,000,000	632 (36.2)
4,000,000 − 5,999,999	618 (35.4)
≥ 6,000,000	495 (28.4)
Education, years	
< 13	428 (24.5)
13 − 14	577 (33.1)
≥ 15	740 (42.4)
Body mass index, kg/m^2^, mean ± SD	21.4 ± 2.8
Daily nutrient intake^c^	
Total energy, kJ, mean ± SD	7434.2 ± 2057.0
Seaweed, g, mean ± SD	12.3 ± 12.7
Fish, g, mean ± SD	46.7 ± 25.8
Yogurt, g, mean ± SD	34.8 ± 39.5

^a^Full-time or part-time employment in the year when the first questionnaire was conducted or in the previous year.

^b^Management; protection services; farming, fishing, or forestry; transportation or communications; or construction.

^c^Nutrient and food intake were adjusted for total energy intake using the residual method.

Table 2 shows the distributions of confounding factors according to seaweed consumption. Seaweed consumption was positively associated with age, professional or technical occupation, and consumption of fish and yogurt and inversely with active smoking status, clerical or related occupation, and sales occupation.

**Table 2:** **Characteristics according to quartile of seaweed consumption in 1745 pregnant women**

	Quartile				
Variable	1 (Lowest) (n = 436)	2 (n = 436)	3 (n = 436)	4 (Highest) (n = 437)	***P***for trend ^a^
Age, years, mean	30.8	31.1	31.6	31.3	0.04
Gestation, weeks, mean	18.5	18.4	18.5	18.7	0.67
Region of residence, %					0.31
Fukuoka Prefecture	56.4	59.2	54.1	52.9	
Other than Fukuoka Prefecture in Kyushu	34.6	30.7	36.0	34.3	
Okinawa Prefecture	8.9	10.1	9.9	12.8	
Number of children, %					0.77
0	39.2	42.0	37.4	42.6	
1	39.2	39.0	44.7	35.2	
≥ 2	21.6	19.0	17.9	22.2	
Nuclear family structure, %	85.8	83.9	85.8	82.4	0.28
History of depression, %	5.5	4.8	4.4	4.6	0.48
Family history of depression, %	10.3	11.5	8.7	9.6	0.45
Having ever smoked,%	37.2	36.0	27.1	28.8	0.0007
Ever experiencing secondhand smoke exposure at home, %	78.2	76.6	72.5	74.1	0.08
Ever experiencing secondhand smoke exposure at work, %	64.9	64.5	62.2	62.0	0.29
Job type^b^, %					0.03
Unemployed	38.1	41.7	40.8	41.0	
Professional or technical	21.1	23.6	28.0	27.0	
Clerical or related occupation	22.7	17.0	17.9	17.6	
Sales	6.0	4.6	5.1	3.4	
Service	6.7	7.6	5.5	6.6	
Production	3.9	3.9	1.6	2.3	
Others^c^	1.6	1.6	1.2	2.1	
Household income, yen/year, %					0.11
< 4,000,000	41.3	35.6	32.3	35.7	
4,000,000 − 5,999,999	33.3	35.8	34.9	37.8	
≥ 6,000,000	25.5	28.7	32.8	26.5	
Education, years, %					0.10
< 13	28.0	23.9	23.2	23.1	
13 − 14	31.9	32.3	36.7	31.4	
≥ 15	40.1	43.8	40.1	45.5	
Body mass index, kg/m^2^, mean	21.7	21.4	21.4	21.4	0.11
Fish, g, mean^d^	40.3	45.7	49.2	51.8	< 0.0001
Yogurt, g, mean^d^	32.4	32.4	36.4	38.1	0.01

^a^For continuous variables, a linear trend test was used; for categorical variables, a Mantel-Haenszel χ^2^-test was used.

^b^Employment status in the year when the first questionnaire was conducted or in the previous year.

^c^Management; protection services; farming, fishing, or forestry; transportation or communications; or construction.

^d^Food intake was adjusted for total energy intake using the residual method.

Table 3 gives ORs and 95% CIs for depressive symptoms during pregnancy by quartiles of seaweed consumption. Compared with seaweed consumption in the lowest quartile, consumption in the second, third, and fourth quartiles was significantly associated with a lower prevalence of depressive symptoms during pregnancy, showing a clear inverse linear trend in crude analysis. After adjustment for age; gestation; region of residence; number of children; family structure; history of depression; family history of depression; smoking; secondhand smoke exposure at home and at work; job type; household income; education; body mass index; and consumption of fish and yogurt, the inverse association was slightly attenuated but remained statistically significant: the adjusted ORs (95% CIs) for depressive symptoms during pregnancy in the first, second, third, and fourth quartiles of seaweed consumption were 1 (reference), 0.72 (0.51 − 1.004), 0.71 (0.50 − 1.01), and 0.68 (0.47 − 0.96), respectively (*P* for trend = 0.03).

**Table 3:** **Odds ratios (ORs) and 95% confidence intervals (CIs) for depressive symptoms during pregnancy by quartiles of seaweed consumption in 1745 pregnant women**

	Quartile				
	1 (Lowest) (n = 436)	2 (n = 436)	3 (n = 436)	4 (Highest) (n = 437)	***P***for trend
Intake, g/day^a^	2.4	5.6	12.6	28.6	
Depressive symptoms, %^b^	24.8	18.8	17.0	16.5	
Crude OR (95% CI)	1.00	0.70 (0.51 − 0.97)	0.62 (0.45 − 0.86)	0.60 (0.43 − 0.83)	0.002
Adjusted OR (95% CI)^c^	1.00	0.72 (0.51 − 1.004)	0.71 (0.50 − 1.01)	0.68 (0.47 − 0.96)	0.03

^a^Values for consumption are medians for adjusted energy intake using the residual method for each quartile.

^b^Prevalence of depressive symptoms during pregnancy based on the Center for Epidemiologic Studies Depression Scale for each quartile.

^c^Adjustment for age; gestation; region of residence; number of children; family structure; history of depression; family history of depression; smoking; secondhand smoke exposure at home and at work; employment; household income; education; body mass index; and intake of fish and yogurt.

## Discussion

The current cross-sectional study revealed that, after adjustment for potential dietary and non-dietary confounding factors, higher seaweed consumption was independently associated with a lower prevalence of depressive symptoms during pregnancy. To the best of our knowledge, this study is the first to demonstrate the association between seaweed consumption and depressive symptoms.

The observed inverse association between seaweed consumption and depressive symptoms may be attributable to the neuroprotective properties of seaweed [1]. Yan *et al.* reported that Hijiki (*Hijikia fusiformis*) showed the strongest 1-diphenyl-2-picryl hydrazyl radical scavenging activity, followed by Wakame (*Undaria pinnatifida*), and that fucoxanthin, a major antioxidant, was the richest carotenoid in Hijiki [20]. Sangeetha *et al.* demonstrated that fucoxanthin had greater potential than β-carotene to modulate lipid perioxidation as well as the activity of catalase and glutathione transferase in the plasma and livers of retinol-deficient rats [21]. With respect to the anti-inflammatory properties of seaweed, dieckol, a compound isolated from the marine brown alga *Ecklonia cava*, significantly inhibited the release of nitric oxide, prostaglandin E_2_, interleukin-1β, and tumor necrosis factor-α in lipopolysaccharide-stimulated murine BV2 microglial cells via the down-regulation of nuclear factor-κB and p38 mitogen-activated protein kinase activation [22]. Alternatively, the other ingredients in seaweed may be preventive against depressive symptoms. For example, a significant inverse association between calcium intake and depression was found in two studies of Korean women [23, 24]. In the present study’s population, higher calcium intake was likewise significantly inversely related to the prevalence of depressive symptoms during pregnancy [19]. A systematic review concluded that higher magnesium intake also seemed to be associated with lower depressive symptoms [25].

The overall diet patterns of subjects who regularly eat seaweed may also be responsible for the observed inverse association. In the Japan Public Health Center-based Prospective Study, a prudent dietary pattern characterized by a high intake of vegetables, fruit, soy products, potatoes, seaweed, mushrooms, and fish was significantly associated with a reduced risk of suicide in both men and women [26]. Depression being a major precondition of suicide, this may indicate that diet health is inversely related to the severity of depressive symptoms.

Some methodological limitations of the present study warrant mention. The cross-sectional nature of the present study hampers the drawing of conclusions about causality.

Our DHQ could only approximate consumption and was designed to assess dietary intake for one month prior to completing the questionnaire. The present study subjects participated in the baseline survey at various points between the 5th and 39th week of pregnancy. The possibility of non-differential exposure misclassification would have biased the estimates of the observed association towards the null.

Depressive symptoms were assessed using the CES-D scale rather than structured diagnostic interviews. The CES-D includes questions on physical symptoms such as fatigue and physical discomfort, which are also typical complaints of pregnancy; the consequence of this symptom overlap could have been an overestimation of depression. The prevalence of depressive symptoms in this study was, however, lower than that in a representative sample of the Japanese general population: the prevalence of depressive symptoms (CES-D score of ≥ 16) was 30.7% in 2315 women aged 30–39 years [27]. Again, our study subjects took part in the baseline survey at various points between the 5th and 39th week of pregnancy. Therefore, it is difficult to accurately estimate the incidence and prevalence of depressive symptoms during pregnancy. The possibility of non-differential outcome misclassification would have given rise to an underestimation of our results.

We could not calculate the participation rate because we do not have exact figures for the number of pregnant women who were provided with a set of leaflets explaining the KOMCHS, an application form, and a self-addressed and stamped return envelope by the 423 collaborating obstetric hospitals. We were not able to assess the differences between participants and non-participants because information on personal characteristics such as age, socioeconomic status, and history of depression was not available for non-participants. Our subjects were probably not representative of Japanese women in the general population, however. For example, a population census conducted in 2000 in Fukuoka Prefecture found that the percentages of women aged 30 to 34 years with < 13, 13–14, ≥ 15, and an unknown number of years of education were 52.0%, 31.5%, 11.8%, and 4.8%, respectively [28]. The corresponding figures for this study were 24.5%, 33.1%, 42.4%, and 0.0%, respectively. Thus, our study subjects were more educated and probably more aware of health topics than women in the general population.

Although adjustment was made for several dietary and non-dietary confounding factors, residual confounding effects could not be ruled out.

## Conclusion

The current cross-sectional study in Japan found that higher seaweed consumption is independently associated with lower prevalence of depressive symptoms during pregnancy. Seaweed is a popular traditional food that is widely consumed, irrespective of age or sex, in Japan. Further studies are necessary to confirm the beneficial effects of seaweed on depressive symptoms.

## Abbreviations

CES-D: Center for Epidemiologic Studies Depression Scale
CI: Confidence interval
DHQ: Diet history questionnaire
KOMCHS: Kyushu Okinawa Maternal and Child Health Study
OR: Odds ratio.
